# Depressive and Anxiety Symptoms Among People Under Quarantine During the COVID-19 Epidemic in China: A Cross-Sectional Study

**DOI:** 10.3389/fpsyt.2021.566241

**Published:** 2021-02-15

**Authors:** Tong Yan, Wang Zhizhong, Zheng Jianzhong, Ying Yubo, Liu Jie, Zhang Junjun, Liu Guangtian

**Affiliations:** ^1^Department of Social Medicine, School of Public Health, Shanxi Medical University, Taiyuan, China; ^2^Department of Epidemiology and Health Statistics, School of Public Health at Guangdong Medical University, DongGuan, China; ^3^Department of Epidemiology and Health Statistics, School of Public Health and Management, Ningxia Medical University, Yinchuan, China; ^4^Department of Preventive Medicine, Chang Zhi Medical College, Changzhi, China; ^5^Department of Infectious Disease, The Fourth People Hospital of Ningxia, Yinchuan, China

**Keywords:** COVID-19, quarantine, depression, anxiety, cross-sectional study

## Abstract

**Background:** During the coronavirus disease 2019 (COVID-19) pandemic, quarantine as an effective public health measure has been widely used in China and elsewhere to slow down the spread, while high-risk psychological response populations remain under-reported.

**Objective:** The aim of the study is to investigate the depressive and anxiety symptoms among the high-risk individuals quarantined during the COVID-19 pandemic in China.

**Methods:** An online survey was conducted from February 29 to April 10, 2020, among individuals quarantined for at least 2 weeks due to the high-risk exposure. Chinese versions of the nine-item Patient Health Questionnaire (PHQ-9) with a seven-item Generalized Anxiety Disorder (GAD-7) were applied to assess depressive and anxiety symptoms, respectively. Compliance with quarantine and knowledge of COVID-19 was also assessed. An unconditional logistic regression model was performed to identify the correlators.

**Results:** Of the 1,260 participants completing the full survey, 14.0% (95% CI: 12.2–16.1%), 7.1% (95% CI: 5.9–8.7%), and 6.3% (95% CI: 5.1–7.8%) had at least moderate symptoms of depression, anxiety, and a combination of depression and anxiety (CDA), respectively; 14.8% (95% CI: 13.0–16.9%) had at least one condition. Multivariate analysis showed that participants with an undergraduate or above degree were more likely to report depressive (OR = 2.98, 95% CI: 1.56–5.72) and anxiety symptoms (OR = 2.95, 95% CI: 1.14–7.63) than those with middle school education. Those who were unemployed (OR = 0.37, 95% CI: 0.21–0.65 for depression; OR = 0.31, 95% CI: 0.14–0.73 for anxiety), students (OR = 0.14, 95% CI: 0.04–0.48 for depression; OR = 0.11, 95% CI: 0.01–0.85 for anxiety), and more knowledgeable of COVID-19 (OR = 0.84, 95% CI: 0.73–0.96 for depression, OR = 0.82, 95% CI: 0.68–0.98 for anxiety) were less likely to report depressive and anxiety symptoms. Higher quarantine compliance correlated with lower risks of depressive (OR = 0.94, 95% CI: 0.91–0.96) and anxiety symptoms (OR = 0.95, 95% CI: 0.91–0.98).

**Conclusion:** Individuals under quarantine during the COVID-19 pandemic suffered prevalent depressive and anxiety symptoms. Consequently, comprehensive interventional measures, including knowledge dissemination, timely virus tests, and strengthened communication, may minimize quarantine's adverse effects.

## Introduction

A cluster of pneumonia cases of coronavirus disease 2019 (COVID-19) was first detected in Wuhan City in December 2019. The number of patients affected with COVID-19 drastically increased throughout the nation in a month, followed by geographical expansion of the spread worldwide. On March 11, 2020, the World Health Organization (WHO) assessed the international public health emergency regarding COVID-19 and characterized it as a pandemic (1). Up to May 20, there were 4,789,205 confirmed cases, and 318,789 deaths due to COVID-19 have been reported from 216 countries, areas, or territories worldwide (2). Additionally, the transmission classifications in most countries were clusters of cases and community transmission.

Although substantial knowledge gaps regarding COVID-19 remain, increasing evidence suggests that efficient person-to-person transmission of COVID-19 occurs, even during the incubation period, in asymptomatic individuals (3–5). Public health measures, including quarantine, isolation, and case tracking, might be the practical tools to contain the virus spread before a preventive vaccine and specific treatment are available (6). The WHO, combined with various bodies, recommends guidelines for stopping the spread under different situations, including at home, during or after travel, in the workplace, etc. (7, 8). Unprecedented quarantine measures have been taken during the COVID-19 pandemic in China. Those individuals with a history of travel to or residence in high-risk areas or countries (where continuous transmission of local cases has been identified) were forced to apply 14 days of self-isolation in dedicated facilities (9).

Quarantine means separation and restriction of movement of persons who have potentially been exposed to a contagious disease but are seemingly healthy (10). As early as the year 1127, quarantine was used in Venice, Italy, to control leprosy. Community- and city-wide quarantine was also imposed during the severe acute respiratory syndrome (SARS) epidemic in the year 2003, and during the outbreak of the Ebola and the Middle East Respiratory Syndrome (MERS) in very recent years. These measures played an essential role in controlling public health events (11).

In China, all individuals are monitored for temperature, and any flu-like symptoms during the quarantine period are separated from their family members and follow other quarantine measures. However, a previous study has shown that the loss of freedom, uncertainty over the possibility of being infected, boredom, and social stigma caused by quarantine may have a psychological impact (11). Mental health evaluation for individuals quarantined in the past epidemic of infectious disease epidemics has revealed that these individuals are more likely than the general population to experience depressive, anxiety symptoms, posttraumatic stress symptoms, and emotional exhaustion (12–14). Longitudinal studies among the general population suggested that depression, anxiety, and stress in response to COVID-19 is not just an initial reaction but potentially the start of a persistent problem that extends beyond the pandemic (15, 16).

During the COVID-19 pandemic, many research studies have reported the mental health of medical health workers, college students, and the general community residents (17–20). However, few studies evaluated mental health outcomes in quarantined persons in the context of COVID-19, and the correlation between psychological response and behavioral compliance toward quarantine and knowledge of COVID-19 in the quarantined population is still under-reported. The present study aimed to investigate the depressive and anxiety symptoms among the high-risk population quarantined during the COVID-19 pandemic in China and identify the correlators. We hypothesized that depressive and anxiety symptoms were prevalent in individuals during the quarantine, and compliance toward quarantine, knowledge of COVID-19, and some other variables may correlate with psychological response among people under quarantine due to COVID-19.

## Materials and Methods

### Participants and Procedure

A cross-sectional study was performed via an online survey through a platform (https://www.wjx.cn/app/survey.aspx) from February 29 to April 10, 2020. Individuals who had a travel history to high-risk areas or countries and placed under mandatory quarantine in Ningxia Province, China, were eligible for this study's potential participation. The exclusion criteria included individuals who could not access the Internet or other mobile devices due to vision or other disabilities leading to an inability to finish the online questionnaire.

An invitation letter was sent to all the possible participants through WeChat (the most popular social media app in mainland China, with 1 billion daily active users). The research team, who provided medical care in the quarantine facility, provide scanning QR codes to access the online survey after completing the informed content. The survey took approximately 8–15 min. In total, 1,385 eligible participants agreed to participate in the survey. After removing the participants with missing values in the mental health outcome measures, data of 1,260 participants were included in the final analysis.

### Measurements

#### Depressive and Anxiety Symptoms

Chinese versions of the nine-item Patient Health Questionnaire (PHQ-9) (21) and the seven-item Generalized Anxiety Disorder Scale (GAD-7) (22) were used to assess the depressive and anxiety symptoms, respectively. These two brief screening instruments have been widely used in medical and community settings to screen, diagnose, monitor, and measure depression and anxiety severity. Each item, rated on a four-point scale from 0 (Not at All) to 3 (Nearly Every Day), measures the frequency of depressive and anxiety symptoms in the last 2 weeks. The PHQ-9 has the total scores categorized as follows: minimal/normal (0–4), mild (5–9), moderate (10–14), and severe (15–27) (21). The GAD-7 has the total scores categorized as mild (5–9), moderate (10–14), and severe (15–21) (22). The Chinese versions of the PHQ-9 and GAD-7 both have strong internal and test–retest reliability as well as construct and factor structure validity in patients and the general population (23, 24). Previous studies have defined a cut-off point of 10, an optimal algorithm scoring method, to detect depression and anxiety symptoms, respectively (25, 26). In this sample, the Cronbach's alpha values for the PHQ-9 and GAD-7 were 0.94 and 0.95, respectively.

#### Behavioral Compliance Toward Quarantine Measures

Compliance during the quarantine period was assessed by asking, “Do you think these quarantine measures (such as remaining inside a room alone, measuring temperature twice daily, or wearing a mask when contact with others in the same space) are necessary?” The five-point Likert scoring response was, very unnecessary (one point), unnecessary (two points), undecided (three points), necessary (four points), and very necessary (five points). There were seven questions; the total score could range from 7 to 35, and higher scores indicated higher compliance with the quarantine measures. The full questionnaires are shown in Supplementary Table 1.

#### The Knowledge of and Attitudes Toward COVID-19

A 10-question questionnaire (developed by epidemiologists and clinicians from two universities and a designated hospital) was used to measure the knowledge of and attitude toward COVID-19 according to the guidelines for the diagnosis and treatment of COVID-19 (standard version) (27). Supplementary Table 2 shows that these questions mainly consisted of epidemiological characteristics, suspected symptoms, and personal protection measures regarding COVID-19. A correct answer recorded one point, and an incorrect/unknown answer recorded zero points. The higher score indicated a better knowledge of COVID-19. Two questions measured attitudes toward COVID-19: “Do you worry about being infected with COVID-19?” and “Do you agree with that for the final control of COVID-19, humans will win the battle against COVID-19?”

### Statistical Analysis

Data analyses were performed using SPSS statistical software (version 22.0, IBM Corp), and *p*-values ≤ 0.05 were considered statistically significant with a two-tailed test. Categorical variables are presented as frequencies and percentages, while continuous variables are presented as the means and standard deviations with ranges. The percentage differences in depressive or anxiety symptoms across categorical variables were examined using the chi-squared tests. Spearman correlation coefficients were used to investigate the correlations between the PHQ-9 and GAD-7 scores. An unconditional regression model was performed to identify the correlators of mental health outcomes after controlling for covariates. The adjusted odds ratios (ORs) and their 95% confidence intervals (95% CIs) of the independent variables were calculated.

## Results

### Demographic Characteristics

The participants finished the survey on the 13th or 14th day of the quarantine or within 1 week after the end of the quarantine. The median time from the start of quarantine to completing the survey was 14 days (interquartile range, 13–18 days). As shown in Table 1, more than half of the participants were male (56.2%), were aged 31–50 years (57.1%), and were living in the urban area (57.9%). Approximately one-third of the participants (34.3%) had an educational level of undergraduate or above. Most participants were married (73.7%) and had been employed (65.6%).

**Table 1:** Demographic characteristics and the scores of knowledge toward COVID-19 and compliance with quarantine measures of participants.

**Variables**	***N* (%)**	**Knowledge toward COVID-19,** **M ± SD**	**Compliance with quarantine measures,** **M ± SD**
Overall	1,260 (100)	8.11 ± 1.26	29.60 ± 5.39
**Gender**			
Male	708 (56.2)	8.05 ± 1.30	29.71 ± 5.36
Female	552 (43.8)	8.19 ± 1.21	29.47 ± 5.43
**Age**			
18–25	225 (17.9)	8.10 ± 1.32	31.25 ± 4.61
26–30	207 (16.4)	7.98 ± 1.33	28.84 ± 6.31
31–40	398 (31.6)	8.12 ± 1.22	29.49 ± 5.22
41–50	321 (25.5)	8.20 ± 1.21	29.19 ± 5.43
≥51	109 (8.7)	8.05 ± 1.31	29.26 ± 4.88
**Education level**			
Middle school	330 (26.2)	7.84 ± 1.29	30.21 ± 4.62
High school	260 (20.6)	7.98 ± 1.38	28.87 ± 5.78
Junior college	238 (18.9)	8.18 ± 1.25	29.57 ± 5.36
Undergraduate and above	432 (34.3)	8.27 ± 1.15	29.60 ± 5.39
**Marriage**			
Unmarried	331 (26.3)	8.09 ± 1.30	30.43 ± 5.37
Married^a^	929 (73.7)	8.11 ± 1.25	29.31 ± 5.37
**Occupation**			
Employed	827 (65.6)	8.12 ± 1.23	29.33 ± 5.57
Unemployed/retired	338 (26.8)	8.03 ± 1.32	29.65 ± 5.29
Students	95 (7.5)	8.24 ± 1.31	31.78 ± 3.30
**Place of residence**			
Urban	729 (57.9)	8.18 ± 1.23	29.36 ± 5.52
Rural	531 (42.1)	8.01 ± 1.30	29.93 ± 5.20
**Worried about being infected**			
Yes	145 (11.5)	8.26 ± 1.06	29.22 ± 5.09
No	1,115 (88.5)	8.09 ± 1.28	29.65 ± 5.43
**Worried about the epidemic can not be controlled**			
Yes	36 (2.9)	8.06 ± 1.41	28.58 ± 7.40
No	1,224 (97.1)	8.11 ± 1.26	29.63 ± 5.32

*M, mean; SD, standard deviation*.

a *Married including divorced and widowed respondents*.

### Knowledge Scores and Attitudes Toward COVID-19

The mean score for knowledge toward COVID-19 was 8.11 ± 1.26 (range:3–10); the accuracy rate for each question on the COVID-19 knowledge questionnaire was 40.6–99.9%. The three questions with the lowest accuracy rates were as follows: the primary infection source was the patients who had been infected by the COVID-19 (40.56%); the main clinical symptoms of COVID-19 are fever, fatigue, dry cough, dyspnea, with or without nasal congestion, runny nose, or other upper respiratory symptoms (57.06%); and the main route of transmission of COVID-19 is respiratory droplet transmission, and it can also be transmitted through contact (71.83%). As shown in Table 1, females had a higher score for knowledge toward COVID-19 than males (8.19 ± 1.21 vs. 8.05 ± 1.30, respectively, *P* = 0.045), and individuals living in urban areas had higher scores than those living in rural areas (8.18 ± 1.23 vs. 8.01 ± 1.30, respectively, *P* = 0.014). The vast majority of the participants did not worry about being infected with COVID-19 (88.5%), and nearly all individuals had confidence that the spreading of the virus can ultimately be controlled (97.1%).

### Compliance Scores Regarding the Quarantine Measures

The mean compliance score for the quarantine measures was 29.60 ± 5.39 (range: 7–35). Most quarantined persons held that measuring temperature twice daily (67.9%), self-health monitoring (65.6%), remaining inside a room alone (62.1%), preventing the sharing of cutlery, towels, or drinking cups (61.5%), and washing hands frequently (55.3%) were necessary. Meanwhile, 42.1% insisted on opening the windows often, and 46.7% continued to wear a mask when in contact with others in the same space. The compliance score regarding the quarantine measures among different characteristic populations is shown in Table 1. The 18–25 age group had higher compliance scores than the older age group (*P* < 0.001). The compliance scores in students were significantly higher than in other groups (*P* < 0.001).

### Depressive and Anxiety Symptoms

As shown in Table 2, the mean scores on the PHQ-9 and GAD-7 in the total sample were 3.76 ± 5.19 (range: 0–27) and 2.64 ± 4.01 (range: 0–21), respectively. Approximately one-third had mild to severe depressive symptoms, whereas the proportions of mild, moderate, and severe depressive symptoms were 17.3, 9.1, and 4.9%, respectively. Nearly one-quarter of the participants had mild to severe anxiety symptoms, and the proportions of those with mild, moderate, and severe anxiety symptoms were 17.7, 5.0, and 2.1%, respectively. The PHQ-9 scores were strongly correlated with GAD-7 scores (*r* = 0.825, *P* < 0.001).

**Table 2:** The severity categories of depression and anxiety symptoms in a quarantined population (*n* = 1,260).

	**Scores M ± SD (range)**	**The severity of the symptoms**, ***n*****(%)**			
		**Normal**	**Mild**	**Moderate**	**Severe**
Depression (PHQ-9)	3.76 ± 5.19 (0–27)	865 (68.7)	218 (17.3)	115 (9.1)	62 (4.9)
Anxiety (GAD-7)	2.64 ± 4.01 (0–21)	948 (75.2)	223 (17.7)	63 (5.0)	26 (2.1)

*M, mean; SD, standard deviation*.

According to the criteria (PHQ-9 ≥ 10, GAD-7 ≥ 10), the percentage of participants in the total sample with depressive and anxiety symptoms was 14.0% (95% CI: 12.2–16.1%) and 7.1% (95% CI: 5.9–8.7%), respectively. The percentage of individuals with at least one condition (anxiety or depression) was 14.8% (95% CI: 13.0–16.9%). The percentage of individuals with both depression and anxiety was 6.3% (95% CI: 5.1–7.8%). As shown in Table 3, the individuals with depressive and anxiety symptoms were associated with lower behavioral compliance scores (29.93 ± 5.24 vs. 27.57 ± 5.87, *P* < 0.001 for depression; 29.76 ± 5.34 vs. 27.57 ± 5.71, *P* < 0.001 for anxiety).

**Table 3:** Percentage of depression and anxiety symptoms among participants with different characteristics (*n* = 1,260).

**Variables**	**Category**	**Depression**			**Anxiety**		
		***N* (%)**	**95% CI^a^**	***P* value**	***N* (%)**	**95% CI^a^**	***P* value**
Total		177 (14.0)	12.2–16.1		90 (7.1)	5.9–8.7	
Gender	Male	98 (13.8)	11.5–16.6	0.812	50 (7.1)	5.4–9.2	0.900
	Female	79 (14.3)	11.6–17.5		40 (7.3)	5.4–10.8	
Age	≤25	26 (11.6)	8.0–16.4	0.665	12 (5.3)	3.1–9.1	0.783
	26–30	39 (18.8)	14.1–24.7		24 (11.6)	7.9–16.7	
	31–40	54 (13.6)	10.5–17.3		24 (6.0)	4.1–8.8	
	41–50	47 (14.6)	11.2–18.9		22 (6.9)	4.6–10.2	
	≥51	11 (10.1)	5.7–17.2		8 (7.3)	3.8–13.8	
Education level	Middle school	18 (5.5)	3.5–8.5	<0.001	7 (2.1)	1.0–4.3	<0.001
	High school	26 (10.0)	6.9–14.3		12 (4.6)	2.7–7.9	
	Junior college	42 (17.6)	13.3–23.0		22 (9.2)	6.2–13.6	
	Undergraduate and above	91 (21.1)	17.5–25.2		49 (11.3)	8.7–14.7	
Marriage	Unmarried	40 (12.1)	9.0–16.0	0.231	14 (4.2)	2.5–7.0	0.017
	Married	137 (14.8)	12.6–17.2		76 (8.2)	6.6–10.1	
Occupation^b^	Employed	156 (18.9)	16.3–21.7	<0.001	82 (9.9)	8.1–12.4	<0.001
	Unemployed/Retired	18 (5.3)	3.4–8.3		7 (2.1)	1.0–4.2	
	Students	3 (3.2)	1.1–8.9		1 (1.1)	0.1–5.7	
Residence place	Urban	121 (16.6)	14.1–19.5	0.002	64 (8.8)	6.9–11.1	0.008
	Rural	56 (10.5)	8.2–13.5		26 (4.9)	3.3–7.1	
Worried about being infected	Yes	44 (30.3)	23.5–38.3	<0.001	33 (22.8)	16.7–30.2	<0.001
	No	133 (11.9)	10.2–14.0		57 (5.1)	6.4–9.5	
Worried about the epidemic can not be controlled^b^	Yes	5 (13.9)	6.1–28.7	0.978	3 (8.3)	2.9–21.8	0.739
	No	172 (14.1)	12.2–16.1		87 (7.1)	5.8–8.7	

*CI, confidence interval*.

a *95% CI means 95% confidence interval of the percentage of depression and anxiety symptoms*.

b *Fisher exact test*.

### Correlators of Depressive and Anxiety Symptoms

As shown in Tables 4, 5, the individuals with junior college and undergraduate degrees or above were more likely to experience depressive and anxiety symptoms than those with middle school degrees; those who were unemployed/retired and students were less likely to experience depressive and anxiety symptoms. After controlling for covaries (education level, gender, residence area, and age), those with higher knowledge scores regarding COVID-19 were less likely to have depressive (OR = 0.84, 95% CI: 0.73–0.96) and anxiety (OR = 0.82, 95% CI: 0.68–0.98) symptoms. Higher behavioral compliance scores regarding the quarantine measures were associated with a lower risk of suffering depressive (OR = 0.94, 95% CI: 0.91–0.96) and anxiety (OR = 0.95, 95% CI: 0.91–0.98) symptoms. Compared with those unmarried individuals, the adjusted odds for anxiety were greater among married individuals (OR = 3.19, 95% CI: 1.48–6.87).

**Table 4:** Univariate and multivariate analyses of the depression symptoms among the quarantined population (*n* = 1,260).

**Variables**	**OR (95% CI)**	***P* value**	**aOR^a^ (95% CI)^a^**	***P* value**
**Gender**				
Male	Ref		Ref	
Female	1.04 (0.76–1.43)	0.812	0.89 (0.62–1.26)	0.499
**Age**				
≤25	Ref		Ref	
26–30	1.78 (1.04–3.04)	0.036	0.68 (0.37–1.26)	0.221
31–40	1.20 (0.73–1.98)	0.471	0.63 (0.36–1.21)	0.117
41–50	1.31 (0.79–2.19)	0.298	0.82 (0.45–1.50)	0.522
≥51	0.86 (0.41–1.81)	0.690	0.47 (0.21–1.07)	0.073
**Education level**				
Middle school	Ref		Ref	
High school	2.05 (1.09–3.86)	0.027	1.51 (0.80–2.92)	0.223
Junior college	4.53 (2.53–8.12)	<0.001	2.77 (1.46–5.25)	0.002
Undergraduate and above	4.64 (2.70–7.98)	<0.001	2.98 (1.56–5.72)	0.001
**Occupation**				
Employed	Ref		Ref	
Unemployed/retired	0.24 (0.15–0.40)	<0.001	0.37 (0.21–0.65)	<0.001
Students	0.14 (0.04–0.45)	0.001	0.14 (0.04–0.48)	0.002
**Residence place**				
Urban	Ref		Ref	
Rural	0.59 (0.42–0.83)	0.002	0.78 (0.52–1.18)	0.242
**Worried about being infected**				
Yes	Ref		Ref	
No	0.31 (0.21–0.46)	<0.001	0.35 (0.23–0.54)	<0.001
Knowledge score of COVID-19	0.92 (0.81–1.04)	0.175	0.84 (0.73–0.96)	0.012
Compliance score toward quarantine measures	0.93 (0.91–0.96)	<0.001	0.94 (0.91–0.96)	<0.001

*Ref, Reference category; OR, odds ratio; aOR, adjusted odds ratio; CI, confidence interval*.

a *Adjusted for gender, age, educational level, and residence place*.

**Table 5:** Univariate and multivariate analyses of the anxiety symptoms among the quarantined population (*n* = 1,260).

**Variables**	**OR(95% CI)**	***P* value**	**aOR^a^(95% CI)^a^**	***P* value**
**Gender**				
Male	Ref		Ref	
Female	1.03 (0.67–1.58)	0.900	0.88 (0.55–1.40)	0.585
**Age**				
≤25	Ref		Ref	
26–30	2.33 (1.13–4.79)	0.022	0.81 (0.37–1.82)	0.814
31–40	1.14 (0.56–2.32)	0.720	0.54 (0.24–1.20)	0.541
41–50	1.31 (0.63–2.70)	0.470	0.70 (0.30–1.61)	0.698
≥51	1.41 (0.56–3.55)	0.471	0.66 (0.37–1.16)	0.760
**Education level**				
Middle school	Ref		Ref	
High school	2.23 (0.87–5.75)	0.096	1.66 (0.62–4.45)	0.314
Junior college	4.70 (1.97–11.19)	<0.001	2.73 (1.05–7.02)	0.038
Undergraduate and above	5.9 (2.64–13.21)	<0.001	2.95 (1.14–7.63)	0.025
**Marriage**				
Unmarried	Ref		Ref	
Married	2.02 (1.13–3.62)	0.019	3.19 (1.48–6.87)	0.003
**Occupation**				
Employed	Ref		Ref	
Unemployed/retired	0.19 (0.09–0.42)	<0.001	0.31 (0.14–0.73)	0.007
Students	0.10 (0.01–0.70)	0.021	0.11 (0.01–0.85)	0.034
**Residence place**				
Urban	Ref		Ref	
Rural	0.54 (0.33–0.86)	0.009	0.66 (0.37–1.16)	0.148
**Worried about being infected**				
Yes	Ref		Ref	
No	0.18 (0.11–0.29)	<0.001	0.20 (0.12–0.34)	<0.001
Knowledge score of COVID-19	0.89 (0.76–1.05)	0.173	0.82 (0.68–0.98)	0.031
Compliance score toward quarantine measures	0.94 (0.91–0.97)	<0.001	0.95 (0.91–0.98)	0.003

*Ref, Reference category; OR, odds ratio; aOR, adjusted odds ratio; CI, confidence interval*.

a *Adjusted for gender, age, educational level, and residence place*.

## Discussion

During major infectious disease outbreaks, especially when in the absence of vaccines and specific treatments, quarantine is an essential and efficient preventive public health measure. However, previous studies have found that quarantine is associated with adverse psychological outcomes during the epidemics of SARS (12), Ebola (28), MERS (14), and influenza 2009 (29). Related studies have suggested that a quarantine's psychological impact is substantial, wide-ranging, and long-term suffering (11). To our knowledge, the psychological effects of quarantine during the COVID-19 pandemic on the individuals have not been well reported. The present study found that depressive and anxiety symptoms were prevalent in individuals during the quarantine in China. The findings are consistent with the studies mentioned above (28, 29). This study also provides the primary evidence for improving quarantine strategies and promoting their effectiveness and social acceptability by delivering better health education.

### The Prevalence of Anxiety and Depressive Symptoms

Our findings are consistent with the studies out of China during the COVID-19 pandemic. There are systematic reviews reported that quarantine status is a predictive factor for depressive and anxiety symptoms among the general population. The prevalence of depressive symptoms ranged from 14.2 to 53.5%, and from 6.33 to 50.9% for anxiety symptoms (30–32). In our sample, the prevalence of depressive and anxiety symptoms was within this range. An increased prevalence of depressive symptoms was reported in this quarantined population (14.0%), which was higher than that of the Shenzhen quarantined population (6.21%) (33) and the Vietnamese outpatients (7.44%) (34), but lower than the prevalence of the Spanish population (18.7%) (20). It should be cautious to compare the prevalence among different studies due to the various instruments used. Even when the same scale is used, the researchers adopted different cut-off points. For example, some studies reported participants with scores above the cut-off point (moderate-to-severe symptoms), while others included any participants with mild-to-severe symptoms. Also, it has been proved that the people's mental state is affected by geographical and temporal distributions (35), so differences in the time points and geographical location of mental health assessment may also associate with inconsistency in these results. Several studies have assessed mental health outcomes among community populations and health care workers during the COVID-19 pandemic in China and used the same instruments and cut-off points as ours. Lai et al.'s data from Chinese medical health workers reported a considerable proportion of participants with depressive (50.4%) and anxiety (44.6%) symptoms (17). Zhang et al. found that the prevalence of anxiety and depressive symptoms was 8.5 and 9.5%, respectively, in the general population (36). In this cross-sectional survey, the prevalence of depressive symptoms among high-risk quarantined persons during the COVID-19 epidemic in China was higher than that among the general population and lower than that among health care workers, consistent with several comparative studies reported that depressive and anxiety symptoms arise during the COVID-19 epidemic (33, 37).

### Correlators of the Psychological Response Among the Quarantined Population

The risk of experiencing depressive and anxiety symptoms was associated with some sociodemographic variables among the high-risk quarantined people. Those with an undergraduate education level or above reported the highest percentage of depressive and anxiety symptoms among all education levels, although studies among the general population found that lower education levels are a risk factor of depressive and anxiety symptoms (30–32). The possible explanation may be because individuals with higher educational degrees probably have a more heightened self-awareness of their health (38). Additionally, Zhou et al. had reported that being overloaded was a risk factor for all measured psychological disturbances, including depression and anxiety (39). The highly educated people are more likely to be employed, hold a higher level or more prominent position in companies and organizations, and have more workload. They may worry about delays in work and subsequent deprivation of their income due to quarantine. The participants with jobs have a higher risk than their counterparts. It is worth mentioning that most of the employed individuals in this sample planned to return to work. One study reported that migrant workers experienced the highest psychological distress level among all occupations during the COVID-19 epidemic in China (40). They were also concerned about exposure to the viruses in public transportation when returning to the city where they worked. Those who are married reported a higher risk of anxiety symptoms than unmarried individuals, probably because they were more worried about their children and other family members and wanted to return to their families as soon as possible. However, in general, divorced or widowed persons were more likely to experience depressive and anxiety symptoms, while pregnant women showed less depressive and anxiety symptoms (31, 41). The participants who worried about being infected tended to experience depressive and anxiety symptoms, which is consistent with general population. They may fear being infected or infecting others, and this fear commonly occurs among the high-risk population (42). This might be exacerbated by the participants experiencing some physical symptoms during the quarantine period or being misled by inadequate information received from social media. Therefore, adequate medical resources and as much accurate information as possible during the quarantine period are still needed. The systematic reviews (30–32) reported that the female, younger age group (≤40 years), and living in urban areas have a greater level of anxiety and depressive symptoms, which is inconsistent with our study due to the participants' demographic difference.

This study also found that quarantined persons had higher scores of knowledge of COVID-19 and behavioral compliance toward quarantine measures, and these two factors were associated with psychological outcomes. Most subjects held a positive attitude toward the battle against COVID-19. Education attainment positively correlated with COVID-19 knowledge scores. This finding is consistent with one study that showed community dwellings with a master's degree were more knowledgeable than those who held lower-level degrees (43). Consequently, we controlled for covariates including educational attainment, gender, residence area, and age, and found that individuals with less knowledge about COVID-19 and lower behavioral compliance to quarantine measures were more likely to have depressive and anxiety symptoms.

Knowledge and understanding of the experiences of quarantined persons may contribute to maximizing infectious disease containment and minimizing the adverse effects on those quarantined, their families, and social networks (12).

### The Comorbidity of Depressive and Anxiety Symptoms

Furthermore, the PHQ-9 scores of the quarantined individuals were strongly correlated with their GAD-7 scores. On the one hand, many studies have reported that depressive and anxiety disorders are strictly related and frequently comorbid (38, 44). On the other hand, these two scales were highly correlated owing to a higher-order factor in analytic models, which consists of nonspecific symptoms common to depression and anxiety (45).

### Possible Measures to Mitigate the Psychological Impact of COVID-19

In summary, the present findings suggested that effective efforts to reduce the psychological impact should be put in place as part of the quarantine planning process. First, safe living conditions and adequate supplies are essential. The infrastructure and space of the quarantine facility should be well organized to limit potential transmission. Adequate supplies, including food, water, appropriate accommodation, and personal protective equipment, should be provided in a timely manner. Meanwhile, the quarantine facility should be staffed by health care workers who can monitor physical symptoms and take measures with suspected cases. Second, the dissemination of knowledge and health promotion strategies should be implemented. Targeted and acceptable health education programs will provide individuals with a good understanding of COVID-19 and help them have a good understanding of why they were quarantined and how it will work. Merino et al. found that people in the intrinsic orientation group (meaning those who are taking advantage of confinement to enjoy being with the family, personal development, and so on) show higher levels of psychological well-being and subjective well-being (46). So reinforcing the sense of altruism and cultivating a conscious appreciation of the social and individual values will reduce the mental health effects and improve their compliance (47). Third, timely and accurate examination involving computed tomography (CT) imaging and the nucleic acid test may (48) eliminate their worries and fears. Fourth, improving communication and providing phone-based or online psychological support (49) or appropriate psychological intervention can maintain and promote mental health. Finally, quarantine requires collaborative efforts from multiple organizations and institutions. The quarantine not only needs planning and implementation by health departments and cooperation by a high risk population but also needs reasonable job and social security for quarantined individuals by the government and society.

## Limitations

Some potential limitations may affect the interpretation and generalizability of the results reported here. First, although an online survey is suitable for larger samples and rapid assessment, if the people were too stressed to respond or not interested in this survey, it may have led to response bias and affected the results. Second, we controlled for many covariates in the logistic regression model. Nevertheless, some possible residual confounding may have been caused by unmeasured variables, such as degree of exposure, family members in quarantine, workload, and social support. Finally, due to the cross-sectional design, the causal relationships between variables and mental outcomes cannot be determined. Therefore, the interpretation of those results should be taken cautiously.

## Conclusions

Individuals under quarantine during the COVID-19 pandemic suffered prevalent symptoms of depression and anxiety. Consequently, comprehensive interventional measures, including dissemination of knowledge, timely examination, and strengthened communication, should be built to minimize the quarantine's adverse effects.

## Data Availability Statement

The raw data supporting the conclusions of this article will be made available by the authors, without undue reservation.

## Ethics Statement

The survey was designed as anonymous. An online informed consent was obtained by asking participants to check a box on the device’s screen with the response (I agree to participate in the survey; I do not agree to participate in the survey). If the answer was “I do not agree,” the computer program was immediately and automatically terminated. This study was approved by the institutional review board of Ningxia Medical University (document number: 2020112).

## Author Contributions

WZ, ZJZ, and TY conceptualized and designed the study. TY, WZ, YY, LJ, LG, and ZJJ acquired the subjects and data. TY prepared the manuscript. WZ and ZJZ revised the manuscript for critical intellectual content. All the authors performed the analysis, interpretation of the data and contributed to the article and approved the submitted version.

## Conflict of Interest

The authors declare that the research was conducted in the absence of any commercial or financial relationships that could be construed as a potential conflict of interest.
