# Supplementary material for: Depressive and Anxiety Symptoms Among People Under Quarantine During the COVID-19 Epidemic in China: A Cross-Sectional Study
Source: Front Psychiatry. 2021 Feb 15;12:566241. doi: 10.3389/fpsyt.2021.566241 (PMC7917112; doi:10.3389/fpsyt.2021.566241)
Supplement: Supplementary file 1 [file Table_1.DOCX]

**Table 1 Behavioral compliance toward quarantine measures**

|  | Do you think these quarantine measures below are necessary? | Very unnecessary | Unnecessary | Undecided | Necessary | Very necessary |
| --- | --- | --- | --- | --- | --- | --- |
| 1 | Measure temperature twice daily | 1 | 2 | 3 | 4 | 5 |
| 2 | Self-health monitoring and if had any suspicious symptoms tell the primary healthcare workers | 1 | 2 | 3 | 4 | 5 |
| 3 | Avoid sharing share personal items ( e.g. cutlery, towels or drinking cups) with others | 1 | 2 | 3 | 4 | 5 |
| 4 | Remain inside a room alone | 1 | 2 | 3 | 4 | 5 |
| 5 | Open windows frequently for ventilation | 1 | 2 | 3 | 4 | 5 |
| 6 | Wash hands frequently | 1 | 2 | 3 | 4 | 5 |
| 7 | Wear a mask when contact with others in the same space | 1 | 2 | 3 | 4 | 5 |
