# Supplementary material for: Depressive and Anxiety Symptoms Among People Under Quarantine During the COVID-19 Epidemic in China: A Cross-Sectional Study
Source: Front Psychiatry. 2021 Feb 15;12:566241. doi: 10.3389/fpsyt.2021.566241 (PMC7917112; doi:10.3389/fpsyt.2021.566241)
Supplement: Supplementary file 2 [file Table_2.DOCX]

**Table 2 Questionnaire of knowledge towards COVID-19**

|  | Questions | Options |
| --- | --- | --- |
| 1 | Up to present, the main infection source was the patients  who had been infected by the COVID-19. | True, false, I don’t know |
| 2 | Not all persons will be infected with COVID-19. Only those who are elderly, have chronic illnesses, and are obese are more likely to be infected. | True, false, I don’t know |
| 3 | People who have close contact with someone infected with the COVID-19 should be immediately quarantine in a proper  place. In general, the observation period is 14 days. | True, false, I don’t know |
| 4 | The main route of transmission of COVID-19 is respiratory droplet transmission and it can also be transmitted through contact. | True, false, I don’t know |
| 5 | If you have suspicious symptoms regarding the COVID-19, you taking public transportation to the hospital without a mask and don’t stay away from others. | True, false, I don’t know |
| 6 | The main clinical symptoms of COVID-19 are fever, fatigue, dry cough, dyspnea etc., with or without nasal congestion, runny nose or other upper respiratory symptoms. | True, false, I don’t know |
| 7 | Visitors entering the affected areas,should take routine precautions including avoiding close contacts with people with acute respiratory infection, washing hands frequently and following appropriate coughing etiquette. | True, false, I don’t know |
| 8 | Community population can wear general medical masks to prevent the infection by the COVID-19 virus. | True, false, I don’t know |
| 9 | If you infected with the COVID-19, you can not go to the hospital and can heal yourself. | True, false, I don’t know |
| 10 | There is no specific drug treatment against the COVID-19 currently, but early symptomatic and supportive treatment can help most patients recover from the infection. | True, false, I don’t know |
